# Cat 6 hurricanes have arrived

**DOI:** 10.1073/pnas.2322597121

**Published:** 2024-02-07

**Authors:** Michael E. Mann

**Affiliations:** ^a^Department of Earth and Environmental Sciences, University of Pennsylvania, Philadelphia, PA 16802

For a number of years, I have argued that we are now, thanks to the effects of human-caused warming, experiencing a new class of monster storms—”category 6” hurricanes. That is to say, we are witnessing hurricanes that—by any logical extension of the existing Saffir-Simpson scale—deserve to be placed in a whole separate, more destructive category from the traditionally defined (category 5) “strongest” storms. Up until now, that was really just a matter of opinion (1). There was no peer-reviewed research to justify the assertion. Now there is, with a new article by Wehner and Kossin in PNAS (2) that lays out a rigorous, objective case for expanding the scale to accommodate climate change-fueled tropical cyclones that are qualitatively stronger and more destructive than conventionally defined category 5 storms.

That hurricanes are expected to become stronger with warming is well established. Kerry Emanuel provided an elegant theoretical foundation for understanding the relationship decades ago, demonstrating (3, 4) that a hurricane can be approximated thermodynamically as a “Carnot Engine”—an idealized heat engine that does work through the compression and expansion of an ideal gas. As with the Carnot engine, the thermodynamics governing a hurricane can be viewed as involving heat exchange between two thermal reservoirs—a high-temperature reservoir (the warm tropical ocean surface) and a low-temperature reservoir (the cold tropopause—the boundary between the relatively unstable troposphere within turbulent atmospheric motions take place and the relatively stable stratosphere, which lies about 14 to 16 km above the surface and serves as a cap on vertical tropospheric motions).

**Fig. 1.**
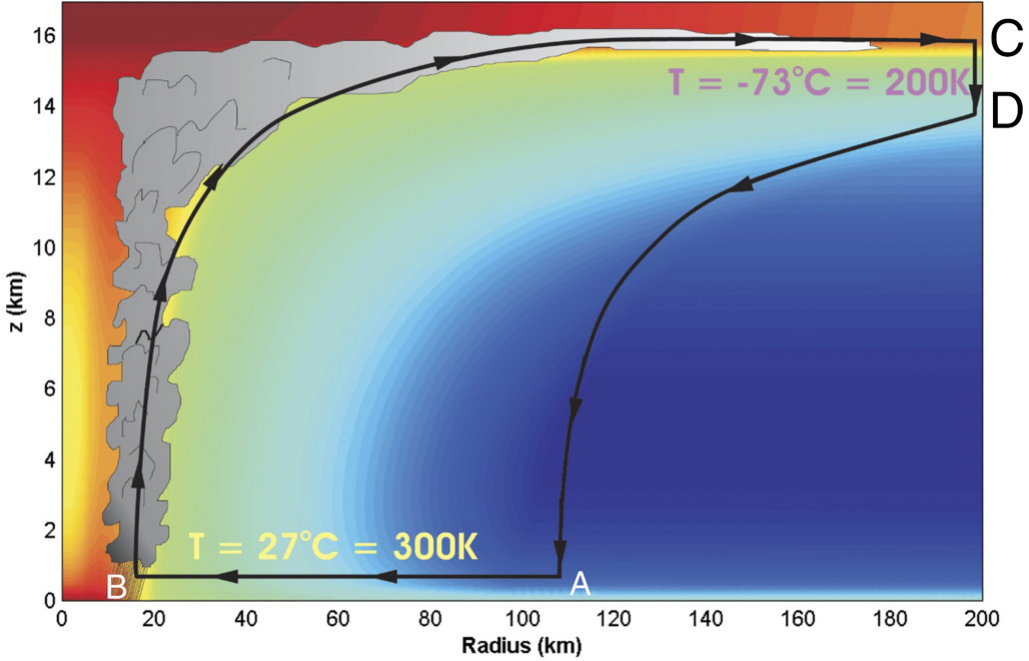
A Hurricane envisioned as a Carnot heat engine (from Emanuel, 2006). This vertical cross-cycle illustrates the thermodynamic cycle discussed in the article (*A-B*) Isothermal Expansion, (*B-C*) Ascension and Adiabatic Expansion, (*C-D*) Isothermal Compression, and (*D-A*) Descension and Adiabatic Compression). The storm center lies along the left edge. Colors depict heat loss (cooler colors) or gain (warmer colors) during the cycle. Reproduced from ref. 4, with the permissions of the American Institute of Physics.

The vertical circulation of winds in the hurricane, in this idealization, is envisioned as consisting (Fig. 1) of two isothermal and two adiabatic or isentropic segments. The inward spiraling and expansion of horizontal surface winds (A to B) occurs at the roughly constant tropical ocean surface temperature, with heat transferred from the ocean to the atmosphere as moisture evaporates at the ocean surface. Surface convergence requires upward vertical motion that takes place within the eye wall, as air rises, expands, and cools roughly adiabatically (B to C; technically the motion is “pseudoadiabatic” because heat is added to the air through the latent heat released when the cooling water vapor condenses into droplets). As the winds spiral out at the tropopause (C to D), undergoing nearly isothermal compression, the air loses the heat gained during ascension through radiation to space. Finally, it undergoes adiabatic compression as it sinks to the surface (D to A), completing a single cycle of the idealized Carnot heat engine.

This conceptual model yields a theoretical relationship between hurricane intensity and surface warming. Conservation of energy requires that heat transferred from the ocean surface to the atmosphere through evaporation must balance the frictional dissipation of kinetic energy associated with surface winds acting on the ocean surface. Emanuel (4) shows that this leads to a proportionality between the squared magnitude of the peak theoretical maximum surface wind *v* (termed the “potential intensity” or “PI”) and the temperature difference (*T_s_*−*T_0_*) between the surface and tropopause, *v*^2^~*T_s_*−*T_0_*. Since the power dissipation (PD) by hurricane surface winds varies as *v*^3^, we have the relationship PD ~ (*T_s_*−*T_0_*)^3/2^ with a proportionality constant that involves *T_0_*and a parameter related to the thermodynamic disequilibrium between the ocean and atmosphere. Under the assumption that both of these latter quantities remain unchanged as the surface warms, we have the result that the squared PI increases linearly with surface warming, and PD increases faster than linearly.

Naturally, like any simple conceptual model, this theoretical description of a hurricane has real-world limitations. The derived expression for PI characterizes the background environmental conditions supporting hurricane development. But for any given storm, other factors can come into play. For example, vertical wind shear (winds that vary in magnitude or direction with height) can interfere with the development of a tropical cyclone, preventing it from forming or reaching its maximum possible strength. Despite these limitations, there are a variety of lines of support for the proposition that human-caused surface warming leads to more intense hurricanes.

State-of-the-art model simulations that are capable of producing realistic hurricanes (5) produce more intense storms with warming. So do dynamical downscaling approaches (6, 7) applied to climate model simulations. Attribution analyses (8), moreover, demonstrate that the overall trend toward increased environmental PI values in recent decades has been driven by human-caused warming (more specifically, greenhouse warming that had remained largely masked by sulfate aerosols until passage of the clean air acts in the 1980s).

Finally, empirical studies (9) bear out the theoretical predictions, demonstrating increasing maximum storm intensity with warming oceans. A roughly 12% increase in wind speed is observed per degree C of warming (roughly the warming the tropical oceans have experienced over the past century) for the strongest (upper decile in intensity) hurricanes. That translates to a roughly 40% increase in destructive potential as measured by PD. That is hardly a modest increase in threat. It should be readily apparent, with serious implications for coastal threat awareness and communication. It is the demonstration that this signal of more intense, more destructive hurricanes has already emerged that motivates Wehner and Kossin’s argument for defining a new “category 6” class of more dangerous and deadly storms.

The tropical storm community—as a whole—has long resisted taking this step. Robert Simpson, coinventor of the scale, once argued that (10) there was no reason to define a higher category than 5 because the damage that can be done saturates at that level: “...when you get up into winds in excess of 155 mph (249 km/h) you have enough damage if that extreme wind sustains itself for as much as 6 s on a building it’s going to cause rupturing damages that are serious no matter how well it’s engineered.” It is possible that argument was once valid, but it is not today thanks to technological progress and more resilient infrastructure in places like southern Florida that are built to withstand category 5 winds (11), defined by sustained winds exceeding 70 m/s/157 mph. Only an extension of the original Saffir–Simpson scale can accommodate these developments.

Extrapolating the existing Saffir–Simpson scale, Wehner and Kossin argue that a storm with sustained winds exceeding 86 m/s/192 mph sustained winds, with substantially greater destructive potential than a conventionally defined category 5 storm, should be defined as a whole new category: category 6.

Extrapolating the existing Saffir–Simpson scale, Wehner and Kossin argue that a storm with sustained winds exceeding 86 m/s/192 mph sustained winds, with substantially greater destructive potential than a conventionally defined category 5 storm, should be defined as a whole new category: category 6. A “cat 6” hurricane, in fact, is hardly just a hypothetical or theoretical construct. The authors note that five storms, all of which have occurred during the past decade—and some of which have proven catastrophic in their impacts—have exceeded that threshold. They include the eastern Pacific hurricane Patricia with its 216 mph peak winds, which made landfall in Jalisco Mexico in October 2015, and four typhoons in the western Pacific (Surigae, 196 mph, which tracked out at sea east of the Philippines in April 2021; Goni, 196 mph, which made landfall in the Philippines in November 2020; Meranti, 195 mph, which impacted Taiwan and the Philippines, landfalling in eastern China in September 2016; Haiyan, 195 mph, with its deadly and devastating landfall in the Philippines in November 2013).

Wehner and Kossin found a significant trend toward more frequent exceedance of cat 6—supporting environmental conditions during the past four decades (1979 to 2019) with a near tripling during the latter half of the interval relative to the first half. That analysis was based on an examination of daily values of environmental PI in modern observational reanalysis data. They also examined state-of-the-art (CMIP6 multimodel intercomparison) simulations both with and without anthropogenic (greenhouse and sulfate aerosol) forcing for that same time interval (bias-corrected based on the reanalysis data). The simulations support a substantial and clearly detectable influence of anthropogenic forcing on the exceedance of cat 6 PI levels. In other words, we expect more cat 6-strength storms in a greenhouse-warmed world. And we are seeing them.

When it comes to evaluating hurricane risk in a warming climate, we should, finally, consider the limitations of the Saffir–Simpson scale in the first place. The scale, for one thing, fails to account for key hurricane impacts other than high winds. Indeed, the majority of hurricane-related fatalities are due to storm surge (~50%) and flooding (~27%). The latter has no bounds. Precipitation rates—and flooding damage and danger—increase exponentially with rising temperatures. The former is a complicated function of a changing climate, as storm surge depends on not just wind speeds but the size of the storm and the speed of forward progress (12). An alternative scale was recently proposed that includes such factors in defining a fiscally based scale for storm surge damage (13).

Such limitations, notwithstanding, Wehner and Kossin argue that “changes in messaging are widely believed necessary to better inform the public” and that while “adding a 6th category to the Saffir-Simpson Hurricane Wind Scale would not solve that issue, it could raise awareness about the perils of the increased risk of major tropical cyclones due to global warming”.

These comments are appropriately measured and subdued for a research article. But I assert the editorial prerogative to be a bit blunter. It is absolutely critical—from a policy standpoint—that the public and policymakers understand the rising coastal threat from more intense, more damaging, and deadly hurricanes. Some members of the tropical storm community have resisted taking such action, calling into question whether or not climate change is impacting tropical storm-related risk (14, 15) or insisting that trends in hurricane activity are due to the putative upturn of a multidecadal natural climate cycle rather than human-caused warming (16). Despite mounting evidence that no such cycle exists (17–19), NOAA’s climate prediction center continues to cite it in their explanations of rising hurricane risk (20). It is time the research and risk assessment and communication community move beyond this outdated thinking and into the 21st century. Lives are literally at stake.
